# Morphological features analysis of eyes with myopic macular retinoschisis in high myopia

**DOI:** 10.1186/s12886-026-05387-3

**Published:** 2026-09-29

**Authors:** Xiaolan Liu, Luyun Liang, Liyan Ye, Yiqin Duan

**Affiliations:** 1 Aier Eye Hospital, Jinan University, N0. 191, Huanshi Middle Road, Yuexiu District, Guangzhou, Guangdong 510071 China; 2 Changsha Aier Eye Hospital, Aier Eye Hospital Group Co., Changsha, Hunan 410015 China

**Keywords:** Myopic macular retinoschisis, Posterior staphyloma, Epiretinal membrane

## Abstract

**Background:**

Myopic macular retinoschisis (MRS) is a vision-threatening disease characterized by macular neuroepithelial layer splitting with or without foveal detachment. Patients with high myopic and posterior staphyloma carry a higher risk of MRS, which may progress to severe vision loss. Epiretinal membrane is a non-vascularized fibrocellular proliferation on the macular inner surface, mainly mediated by Müller cells. Currently, studies on the relationships among MRS, posterior staphyloma and epiretinal membrane are limited, and the role of epiretinal membrane remains underrecognized.

**Methods:**

This retrospective cross-sectional study that included 69 patients (138 eyes) diagnosed with unilateral or bilateral MRS between November 2018 and June 2020 aimed to analyze the complications of MRS and the morphological types of posterior staphyloma in patients with high myopia. Collect clinical data, including age, sex, best corrected visual acuity, axial length, complications of MRS and posterior staphyloma morphology. OCT was used to assess the complications of MRS and B-ultrasound was applied to classify posterior staphyloma. Accompanying conditions, such as epiretinal membrane, macular hole and choroidal neovascularization, were recorded.

**Results:**

A total of 69 patients (138 eyes) were included. Thirty-eight patients (55.1%) had unilateral MRS and 31 (44.9%) had bilateral MRS, resulting in 100 MRS eyes and 38 non-MRS eyes. The median axial length was 30.61 mm (IQRs, 28.71–31.99). Among the MRS eyes, ERM was present in 74 eyes (74.0%). After adjustment for age, sex, axial length, posterior staphyloma and inter-eye correlation, ERM remained significantly associated with MRS (adjusted OR: 4.20, 95% CI: 1.87–9.42, *P* < 0.001). Among the 129 eyes with gradable posterior staphyloma morphology, the fusiform pattern was the most common subtype (57 eyes, 44.2%).

**Conclusions:**

Epiretinal membrane was common in eyes with MRS and seemed to be associated with MRS development. Posterior staphyloma morphology in MRS patients has diverse characteristics.

**Clinical trial number:**

Not applicable.

## Background

Myopic macular retinoschisis (MRS) has been recognized as a significant vision-threatening disorder observed in high myopia (spherical equivalent ≤ -6.00 diopters (D) or axial length ≥ 26.0 mm). Phillips et al. firstly described MRS as a retinal detachment at the posterior pole in highly myopic eyes, occurring in the absence of a macular hole (MH) [1]. With the development of optical coherence tomography (OCT), MRS is now confirmed as a structural separation within the neuroepithelial layer in the macular region, with or without associated foveal detachment [2, 3]. Recent studies have demonstrated that individuals with high myopia and posterior staphyloma (PS) are at an elevated risk of developing MRS, with reported incidence rates ranging from 14.7% to 20% [4, 5]. As the condition progresses, MRS may be complicated by MH formation or macular retinal detachment, which can ultimately result in severe vision loss or blindness without timely surgical intervention [6–8].

Epiretinal membrane (ERM) is a non-vascularized fibrocellular proliferation on the inner surface of the macula, characterized by metamorphopsia and visual impairment [9]. ERM formation is primarily driven by Müller cells, hyalocytes, retinal pigment epithelial (RPE) cells, and myofibroblasts [10–12].

However, as for now, there are few studies concerning the associations among MRS, PS and ERM, and the role of ERM has not been emphasized. Therefore, this study aimed to investigate the complications of MRS and the morphological types of PS in high myopia.

## Methods

### Participants

This was a retrospective and cross-sectional study. A total of 69 patients (138 eyes) diagnosed with unilateral or bilateral MRS at Changsha Aier Eye Hospital between November 2018 and June 2020 were included. Participants were enrolled at the patient level and both eyes were included to analyze bilateral ocular conditions of MRS patients. Inclusion criteria were as follows: (1) at least one eye diagnosed as high myopia that defined as a spherical refractive error ≤ -6.00 D and/or an axial length of ≥ 26.0 mm; (2) significant visual decline or metamorphopsia within the past six months; and (3) a diagnosis of MRS associated with high myopia confirmed by OCT, characterized by interlaminar separation of the retina at the fovea. Exclusion criteria: (1) history of pars plana vitrectomy; (2) history of posterior scleral reinforcement; (3) a history of severe ocular trauma; (4) active uveitis; (5) uncontrolled glaucoma; (6) ocular media opacities; and (7) severe cardiovascular disorders, marked hepatic or renal dysfunction, or other systemic diseases.

### Ophthalmic examinations

All patients received comprehensive demographic and ophthalmologic evaluations, such as age, sex, best corrected visual acuity (BCVA) and axial length. Slit-lamp biomicroscopy and dilated stereoscopic fundus examination were performed for each patient. All eyes underwent OCT (RTVue-XR; Optovue), ultra-widefield fundus imaging (Daytona P200T, Optos), B-scan ultrasonography (AVISO, Quantel Medical) and IOL Master 700 (Zeiss). The complications of MRS were assessed using OCT with cross-sectional scans centered on the fovea. Each scan consisted of 24 radial lines, each 6 mm in length. ERM was defined on OCT as a hyperreflective linear or band-like structure located on the inner retinal surface within the macular region, with or without retinal folds, distortion of the foveal pit, or other tractional changes in the underlying retina [13]. PS was defined as a localized outward protrusion of the ocular wall, with its curvature radius being smaller than that of the adjacent ocular wall [14]. Because the scanning range of OCT in this study was insufficient to comprehensively assess the extent, margins and configuration of posterior staphyloma, all examined eyes were scanned using a 10 MHz probe for B-scan ultrasonography with reference to previously described ultrasonographic methods [15, 16]. The examination was performed with the patient in the supine position, with the eyes closed and without anesthesia. Ultrasound gel was applied to the closed eyelid and the probe was placed on the eyelid. The scanning of the posterior pole of eyeball was performed in horizontal and vertical axial orientations. During the horizontal axial scans, the probe was centered over the cornea with the scanning plane oriented along the 3–9 o’clock meridian and the probe marker was directed nasally; during the vertical axial scans, the probe was centered over the cornea with the scanning plane oriented along the 12–6 o’clock meridian and the marker was directed superiorly. The peripheral retina was routinely evaluated by transverse and longitudinal scans. The clock-hour positions for longitudinal scanning were not predetermined. Instead, longitudinal scanning was performed in a radial manner according to the retinal region requiring further evaluation. At each selected meridian, the probe was perpendicular to the corneoscleral limbus, with the probe marker pointed toward the limbus and corneal center. Suspicious peripheral lesions were generally first localized using transverse scans and then further evaluated with longitudinal scans. For lesions near the anterior segment or equator region, the probe position and angle were adjusted as needed to improve visualization. Ultrasound gain was not routinely increased but was adjusted as necessary according to image quality and the characteristics of the ocular findings. The same standardized scanning protocol was applied to both eyes. The presence of PS and its morphology were determined based on a comprehensive assessment of the horizontal and vertical axial B-scan images. Four PS morphologies in this study were classified based on Chen et al. [15]: (1) oval, characterized by a posterior concavity of the eye wall with a similar local curvature on both horizontal and vertical scans; (2) fusiform, characterized by a narrow and elongated concavity of the posterior eye wall on both horizontal and vertical scans; (3) rectangular, characterized by approximately right-angled transitions between the posterior walls and the lateral walls on both horizontal and vertical scans; (4) conical, characterized by the gradual narrowing of the lateral walls toward the center on both horizontal and vertical scans. Additionally, two other patterns observed in this cohort were categorized as (5) V-shaped, characterized by the central narrowing of the posterior wall on both horizontal and vertical scans, with a smaller angle than the conical shape; (6) W-shaped, characterized by a double-kyphotic bulge on both sides of the optic disc on both horizontal and vertical scans. Eyes without a localized change in the curvature of the posterior wall were classified as no PS. Eyes in which the contour of posterior wall could be adequately assessed, including the six PS patterns and without PS, were considered gradable. Eyes in which the contour could not be evaluated because of silicone oil tamponade were classified as ungradable. The six PS classifications used in this study were based on the morphology of PS rather than the location of staphyloma. The relationship between the position of PS and MRS was not classified independently because the B-scan ultrasonographic images did not provide sufficient comprehensive three-dimensional information for localization. Axial lengths were measured by the IOL Master 700 in six directions. All research procedures were compliant with the ethics guidelines specified in the Helsinki declaration and were approved by the Changsha Aier Eye Hospital’s ethics committee. Written informed consent was obtained from all participants prior to enrollment in the study.

### Statistical analysis

Statistical analyses were performed by Statistical Package for Social Sciences (SPSS Inc., Chicago, USA) version 23.0. Statistical descriptions were made for demographic and clinical variables, such as age, sex, BCVA, axial length and central retinal thickness (CRT). Normal data were expressed as mean ± standard deviation (SD) and non-normal variables data were expressed as median and interquartile ranges (IQRs). The categorical variables were presented as numbers and percentages. Because both eyes from the same participant were included, factors associated with MRS were analyzed using logistic generalized estimating equation (GEE) models to account for the correlation between the two eyes. The odds ratios (OR), 95% confidence intervals (CI) and P-values were reported. At the same time, Gaussian GEE model was used to analyze the association of PS status and morphology with axial length. Bonferroni-adjusted pairwise comparisons were performed for the PS morphological subtypes. Notably, PS status analysis included 135 gradable eyes, whereas morphology analysis was restricted to the 129 gradable eyes with PS. P-value < 0.05 was considered as statistical significance.

## Results

### Basic information

A total of 69 patients (138 eyes) diagnosed with MRS were enrolled in this study. Thirty-eight patients (55.1%) had unilateral MRS and 31 (44.9%) had bilateral MRS. The median age was 56.00 years (IQRs, 49.50–66.50). There were 20 (29.0%) males and 49 (71.0%) females. At the eye level, the median axial length was 30.61 mm (IQRs, 28.71–31.99) and the median BCVA was 0.92 logMAR (IQRs, 0.40–1.75). Additionally, the median CRT was 360.00 μm (IQRs, 253.50-508.50). Detailed patient-level and eye-level characteristics are presented in Tables 1 and 2, respectively.

**Table 1:** Patient-level characteristics of the study population

Characteristics	Total (*n* = 69)
Age, years, median (IQRs)	56.00 (49.50, 66.50)
Male, n (%)	20 (29.0%)
Female, n (%)	49 (71.0%)
Patients with unilateral MRS, n (%)	38 (55.1%)
Patients with bilateral MRS, n (%)	31 (44.9%)

IQRs = interquartile ranges; MRS = myopic macular retinoschisis

Non-normal variables data are expressed as median (IQRs) and categorical variables are presented as n (%)

**Table 2:** Eye-level characteristics of the study population

Characteristics	Total (*n* = 138)	MRS eyes (*n* = 100)	Non-MRS eyes (*n* = 38)
Right eyes, n (%)	69 (50.0%)	49 (49.0%)	20 (52.6%)
AL, mm, median (IQRs)	30.61 (28.71, 31.99)	30.63 (28.93, 31.88)	30.51 (27.41, 32.26)
BCVA, logMAR, median (IQRs)	0.92 (0.40–1.75)	1.00 (0.52, 1.70)	0.70 (0.22, 2.70)
CRT, µm, median (IQRs)	360.00 (253.50, 508.50)	447.50 (305.50, 581.50)	247.50 (227.75, 300.00)
PS, n/N (%)	129/135 (95.6%)	97/99 (98.0%)	32/36 (88.9%)
ERM, n (%)	85 (61.6%)	74 (74.0%)	11 (28.9%)
MH, n (%)	13 (9.4%)	11 (11.0%)	2 (5.3%)
Lamellar MH	10 (7.2%)	10 (10.0%)	0 (0.0%)
Full-thickness MH	3 (2.2%)	1 (1.0%)	2 (5.3%)
CNV, n (%)	14 (10.1%)	9 (9.0%)	5 (13.2%)
FD, n (%)	32 (23.2%)	31 (31.0%)	1 (2.6%)

MRS = myopic macular retinoschisis; AL = axial length; BCVA = best corrected visual acuity; logMAR = logarithm of the minimum angle of resolution; CRT = central retinal thickness; PS = posterior staphyloma; ERM = epiretinal membrane; MH = macular hole; CNV = choroidal neovascularization; FD = foveal detachment

Non-normal variables data are expressed as median (IQRs) and categorical variables are presented as n (%). For PS, data are presented as n/N (%), where n is the number of eyes with PS and N is the number of eyes with gradable B-scan ultrasonographic assessment. Three eyes were ungradable because of silicone oil tamponade and were excluded from the denominator

### OCT-based complications assessment of both eyes in myopic macular retinoschisis patients

Complications in 138 eyes from 69 patients with unilateral or bilateral MRS were assessed using OCT. Among the 69 patients, 31 patients (44.9%) had bilateral MRS and 38 patients (55.1%) had unilateral MRS, resulting in 100 MRS eyes and 38 non-MRS eyes. The most prevalent feature in MRS eyes was ERM, observed in 74 eyes (74.0%). Foveal detachment (FD) associated with MRS was identified in 31 eyes (31.0%). Additionally, lamellar MH associated with MRS was found in 10 eyes (10.0%), while a full-thickness MH with macular fibrosis was identified in only one eye (1.0%). Choroidal neovascularization (CNV) was observed in 9 eyes (9.0%).

**Fig. 1:**
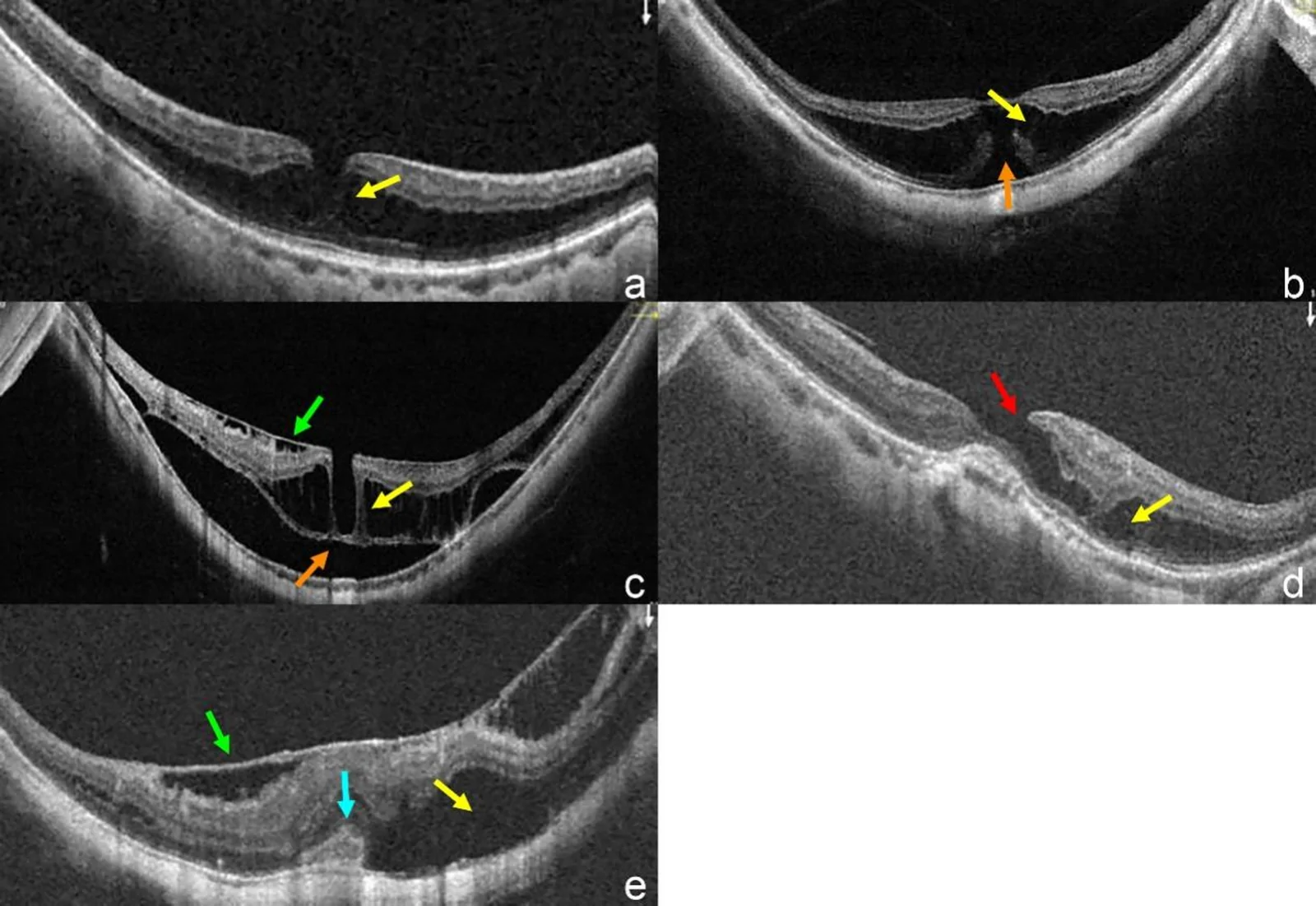
OCT images of MRS complications. (**a**) isolated MRS: the yellow arrowhead shows isolated MRS in the outer retinal layers. (**b**) MRS with FD: the yellow arrowhead shows isolated MRS in the outer retinal layer; the orange arrowhead indicates foveal detachment. (**c**) MRS with ERM and FD: the yellow arrowhead shows isolated MRS in the outer retinal layers; the green arrowhead indicates a thin strip of ERM exerting traction on the neuroepithelium; the orange arrowhead denotes foveal detachment. (**d**) MRS with lamellar MH: the yellow arrowhead shows isolated MRS in the outer retinal layers; the red arrowhead shows a lamellar hole in the foveal area. (**e**) MRS with ERM and CNV: the yellow arrowhead shows isolated MRS in the outer retinal layers; the green arrowhead indicates ERM; the blue arrowhead denotes CNV with a hyperreflective sub-retinal pigment epithelial lesion

Among the 38 non-MRS eyes, the predominant complication was also ERM, observed in 11 eyes (28.9%). Additionally, CNV was identified in 5 eyes (13.2%). Full-thickness MH was present in 2 eyes (5.3%), while no cases of lamellar MH were observed in this subgroup. Further details of complications in both eyes are presented in Table 2 and the OCT imagings of MRS are shown in Fig. 1.

### Morphological classification of posterior staphyloma

**Fig. 2:**
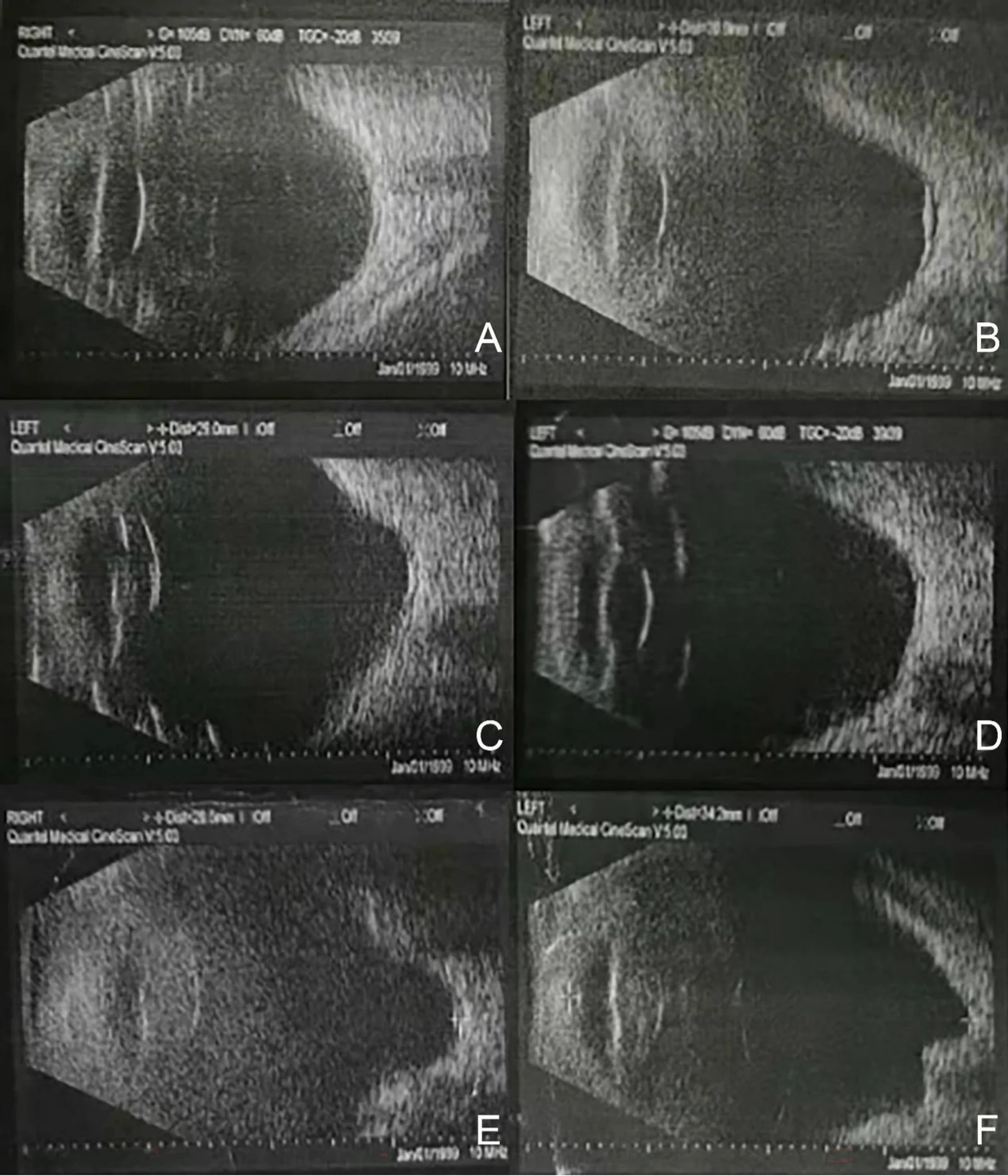
Representative B-scan ultrasonography images of six morphological types of posterior staphyloma. (**A**) Oval pattern, characterized by the posterior wall with a similar local curvature on both horizontal and vertical scans. (**B**) Fusiform pattern, characterized by a narrow and elongated posterior concavity of the posterior wall on both horizontal and vertical scans. (**C**) Conical pattern, characterized by gradual narrowing of the two lateral walls toward the center on both horizontal and vertical scans. (**D**) Rectangular pattern, characterized by approximately right-angled transitions between the posterior walls and the lateral walls on both horizontal and vertical scans. (**E**) V-shaped pattern, characterized by a narrow central apex with a smaller included angle than that of the conical pattern on both horizontal and vertical scans. (**F**) W-shaped pattern, characterized by a double-kyphotic bulge on both sides of the optic disc on both horizontal and vertical scans

The morphology of PS in both eyes of 69 patients with MRS was evaluated by B-scan ultrasonography. Of the 135 eyes with gradable ultrasound images, PS was detected in 129 eyes (95.6%), whereas 6 eyes (4.4%) showed no PS. Among the 129 eyes with PS, fusiform staphylomas were the most common morphological type (57 eyes, 44.2%), followed by oval staphylomas (34 eyes, 26.4%), rectangular staphylomas (21 eyes, 16.3%), and conical staphylomas (9 eyes, 7.0%). Additionally, types W and V staphylomas were found in 5 eyes (3.9%) and 3 eyes (2.3%), respectively. Notably, three eyes showed pseudo-expansion on ultrasound imaging due to silicone oil tamponade, which were classified as ungradable. Detailed distributions of PS morphology according to MRS status are presented in Table 3 and representative ultrasound images are shown in Fig. 2.

**Table 3:** Distribution of posterior staphyloma morphological subtypes according to MRS status

PS morphology	Overall (*n* = 129)	MRS eyes (*n* = 97)	Non-MRS eyes (*n* = 32)
Fusiform, n (%)	57 (44.2%)	44 (45.4%)	13 (40.6%)
Oval, n (%)	34 (26.4%)	24 (24.7%)	10 (31.3%)
Rectangular, n (%)	21 (16.3%)	15 (15.5%)	6 (18.8%)
Conical, n (%)	9 (7.0%)	6 (6.2%)	3 (9.4%)
W-shaped, n (%)	5 (3.9%)	5 (5.2%)	0 (0.0%)
V-shaped, n (%)	3 (2.3%)	3 (3.1%)	0 (0.0%)

PS = posterior staphyloma; MRS = myopic macular retinoschisis

Categorical variables are presented as n (%)

### Factors associated with MRS

In the univariable logistic GEE analysis accounting for inter-eye correlation, ERM was significantly associated with MRS (OR: 4.76, 95% CI: 2.18–10.41, *P* < 0.001), whereas age, sex, axial length, and PS were not significantly associated with MRS. After adjustment for age, sex, and axial length in Model 1, ERM remained significantly associated with MRS (adjusted OR: 4.70, 95% CI: 2.11–10.45, *P* < 0.001). In Model 2 that additionally included PS, the association between ERM and MRS remained significant (adjusted OR: 4.20, 95% CI: 1.87–9.42, *P* < 0.001), whereas PS was not statistically significant (adjusted OR: 2.78, 95% CI: 0.24–31.69, *P* = 0.410) (Table 4).

**Table 4:** Univariable and multivariable logistic GEE analyses of factors associated with MRS

Variables	Univariable Analysis		Multivariable Analysis (Model 1^a^)		Multivariable Analysis (Model 2^b^)	
	OR (95% CI)	*P* value	OR (95% CI)	*P* value	OR (95% CI)	*P* value
Age, per 10-year increase	0.84 (0.64–1.11)	0.224	0.77 (0.57–1.03)	0.076	0.81 (0.60–1.10)	0.183
Sex	0.71 (0.39–1.30)	0.267	0.64 (0.30–1.34)	0.233	0.61 (0.28–1.34)	0.219
AL, per 1 mm increase	1.03 (0.90–1.18)	0.688	1.03 (0.91–1.17)	0.638	1.01 (0.84–1.21)	0.951
ERM	4.76 (2.18–10.41)	< 0.001	4.70 (2.11–10.45)	< 0.001	4.20 (1.87–9.42)	< 0.001
PS	3.75 (0.77–18.36)	0.103	-	-	2.78 (0.24–31.69)	0.410

GEE = generalized estimating equation; AL = axial length; ERM = epiretinal membrane; PS = posterior staphyloma; OR = odds ratio; CI = confidence intervals

^a^ Model 1 includes age, sex, AL and ERM in GEE model

^b^ Model 2 includes age, sex, AL, ERM and PS in GEE model

The statistical significance was considered at a probability of *P* < 0.05

### Relationship of posterior staphyloma status and morphology with axial length

**Fig. 3:**
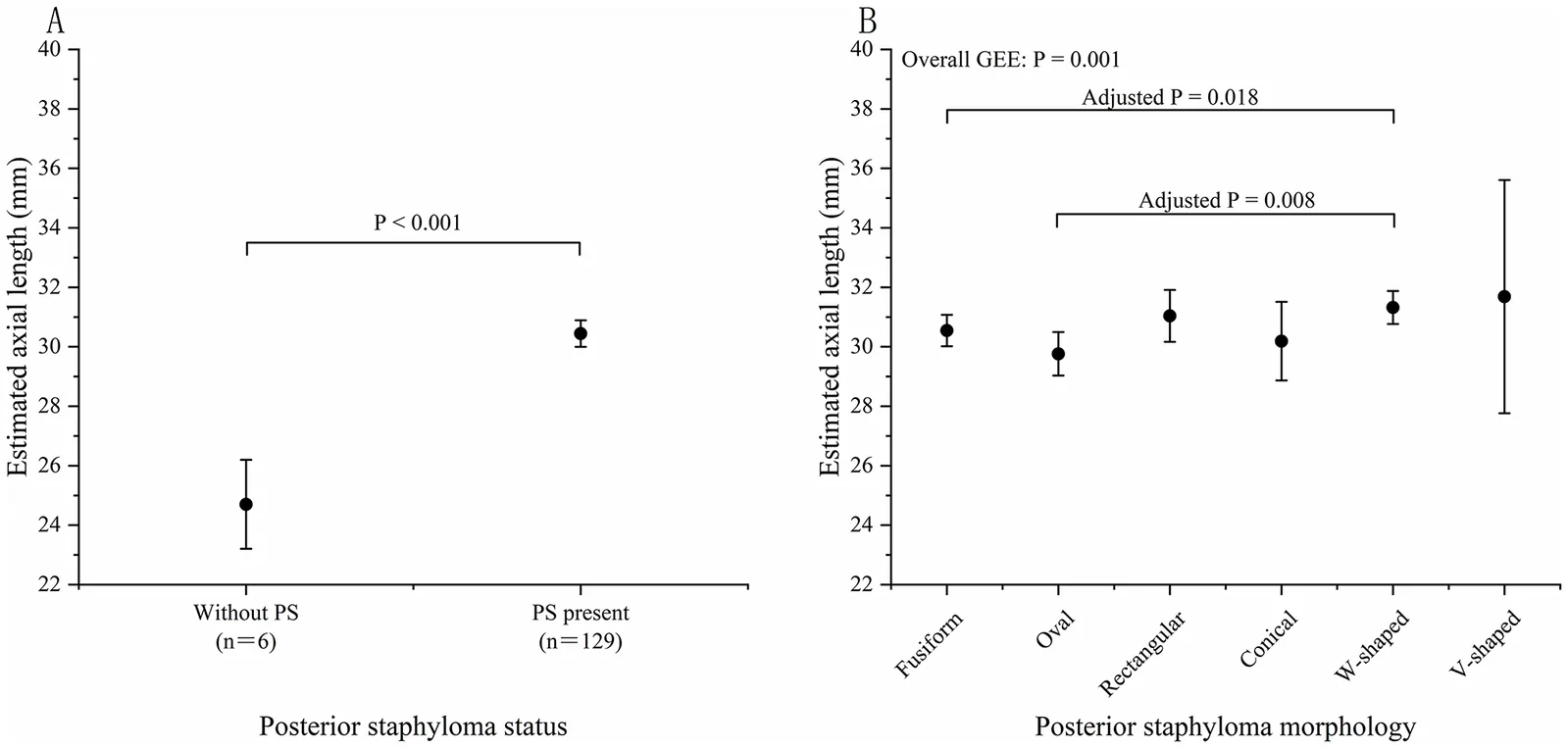
Association of posterior staphyloma status and morphology with estimated axial length. (**A**) Comparison of estimated axial length between without PS and PS present (*P* < 0.001). (**B**) Comparison of estimated axial length between different types of PS morphology (*P*=0.001)

Among the 135 eyes with gradable PS status, eyes with PS had a significantly greater estimated mean axial length than those without PS (mean difference: 5.74 mm, 95% CI: 4.22–7.26, *P* < 0.001). Among the 129 eyes with gradable PS morphology, axial length differed significantly among the six morphological subtypes after accounting for inter-eye correlation (Wald χ² = 19.791, df = 5, *P *= 0.001). Bonferroni-adjusted comparisons showed that the W-shaped pattern had a greater estimated mean axial length than the fusiform pattern (mean difference: 0.77 mm, 95% CI: 0.07–1.48, adjusted *P* = 0.018) and the oval subtype (mean difference: 1.56 mm, 95% CI: 0.23–2.88, adjusted *P* = 0.008). These findings are illustrated in Fig. 3.

## Discussion

MRS, characterized by interlaminar splitting in the macular area, is a common manifestation of myopic traction maculopathy and can contribute to visual impairment in high myopia [17]. Since Takano and Kishi firstly described this tractional lesion in 1999 [2], MRS has gradually been recognized as a combination of biomechanical forces, which include centripetal traction from the posterior vitreous cortex [18], tangential traction force of ERM [19] and the inner limiting membrane [20], and the outward expansion force associated with PS [21]. MRS also may be caused by choroidal atrophy and reduced blood flow to the choroid membrane [22]. It is quite interesting that a dome-shaped macula in eyes of high myopia was associated with a reduced risk of MRS, suggesting that this anatomical configuration may serve as a protective factor [23]. Furthermore, Giancarlo et al. found different surgical responses among MRS subtypes and showed the heterogeneity of this disease [24].

Our study revealed some common complications from OCT imaging in 69 MRS patients. Most MRS patients had one to two or even three other macular complications. Among the 100 MRS eyes, ERM (74.0%) was the most common MRS complication. In terms of non-MRS eyes, ERM remained one of the more common features because it appeared in 11 eyes (28.9%), second only to those without detectable lesion (20 eyes, 52.6%). However, Liu et al. found that CNV had the highest prevalence (41.4%) in the non-MRS group [8], which may contribute to limited sample size and different research cohorts. This discrepancy may stem from the fact that Liu et al.’s study enrolled patients with PS, whereas our cohort specifically consisted of cases diagnosed with unilateral or bilateral MRS.

After accounting for inter-eye correlation, ERM was significantly associated with MRS in the univariable logistic GEE analysis. This association remained significant after adjusting for age, sex, axial length and PS in Model 2. These findings may further support that ERM may play a contributing role in the pathogenesis of MRS [19, 25, 26]. Previous studies have shown that MRS may be influenced by specific inward traction forces, including ERM and vitreoretinal traction, as well as other inward-tending lesions such as retinal vascular microfolds [27, 28]. In addition, we speculate that ERM adhesion may reduce the compliance of the retina and fail to adapt to the morphological changes of PS, resulting in inter-layer separation in the vertical direction. Prolonged action of combined traction force acting on the macula may potentially lead to accelerating the progression of MRS and increasing the risk of severe complications such as MH and FD.

PS has been reported to play a critical role in the pathogenesis of MRS [29]. In a study by Baba et al. [30], OCT was used to examine the macular area of 78 patients (134 eyes) with high myopia. Patients were stratified into groups based on the presence or absence of PS. The results showed that MRS occurred exclusively in eyes with PS, with an overall incidence of 9.0% (7 cases), all found within the PS group (45 eyes). Our findings are consistent with this view: PS was detected in nearly all evaluated eyes except for a few cases obscured by silicone oil tamponade. PS was present in 98.0% of MRS eyes and 88.9% of non-MRS eyes. However, PS was not significantly associated with MRS in either the univariable logistic GEE analysis or the Model 2. The lack of statistical significance may be largely attributed to the small number of eyes without PS, which resulted in imprecise effect estimation and a wide confidence interval, as well as the high prevalence of PS in both the MRS and non-MRS groups, thereby weakening the comparison effect between the two groups. Beyond the presence of PS, the depth of macular staphyloma may provide additional information regarding its relationship with MRS. García-Ben et al. reported that greater macular staphyloma depth was significantly associated with the presence of MRS [31]. Similarly, Park et al. also found that eyes with myopic traction maculopathy had significantly greater staphyloma height and steeper foveal curvature than high myopic eyes [32]. However, PS depth was not quantitatively assessed in our retrospective cohort, resulting in the inability to evaluate this relationship in the present study. Larger prospective studies including more eyes with PS and three-dimensional ocular reconstruction combined with fundus imaging in the future are needed to clarify the independent contribution of PS to MRS development.

After Antonio Scarpa [33] discovered the first PS case by postmortem examination in 1801, Curtin et al. [34] conducted indirect fundoscopy in 250 patients with high myopia and divided PS into ten categories. Ohno-Matsui et al. [35] used three-dimensional magnetic resonance imaging (3D MRI) to construct 3D ocular models in 105 patients (198 eyes) with high myopia. Based on Curtin’s research, they divided PS into six types: wide macular staphyloma, narrow macular staphyloma, peripapillary staphyloma, nasal staphyloma, inferior staphyloma, and a group of others. Among the 198 eyes studied, 100 (50.5%) eyes had PS, with wide macular staphyloma being the most prevalent subtype (74%). Although 3D MRI provides a more comprehensive evaluation of PS morphology in high myopic eyes, it cannot be widely used in clinical practice as a screening method for patients with high myopia due to its high cost and complicated operation.

By contrast, B-ultrasound is low-cost and widely accessible, making it a practical tool in clinical practice as a first-line imaging method in ophthalmology. Chen et al. identified four PS types in highly myopic eyes by B-scan imaging, with the rectangular pattern being the most common [15]. In our study, six subtypes of PS were observed in 129 eyes from MRS patients. Fusiform type was the most frequent, followed by oval, rectangular, and conical types, with the V-shaped and W-shaped patterns occurring less commonly. Differences between our findings and those of Chen et al. may stem from differences in study populations and the interpretation of B-scan contours. Their study mainly included high myopic eyes, whereas our cohort only consisted of MRS patients. Given that the incidence of MRS in high myopia was approximately 50% [35], variations in PS subtype distribution between the two cohorts are expected.

Based on B-ultrasound imaging, the arcuate type described by Chen et al. was similar to the oval type in our study, while both the wedge types and the rectangular types seem to correspond to our rectangular type. The identification of W and V types in our study may reflect greater morphological heterogeneity of PS in patients with MRS compared to those with high myopia alone. Although the W-shaped and V-shaped patterns observed in our study may show similar morphologies to the peripapillary and narrow macular types described in previous location-based classification respectively, these proposed correspondence remains speculative and unverified. Since the location of PS was not assessed in this study, the W-shaped and V-shaped patterns should be regarded as contour-based morphological categories rather than equivalents of established topographic subtypes. PS status was significantly associated with axial length (*P* < 0.001). Axial length also differed significantly among the six PS subtypes (Wald χ²=19.791, df = 5, *P* = 0.001). Bonferroni-adjusted pairwise comparisons showed that the W-shaped subtype had a greater estimated axial length than the fusiform subtype (adjusted *P* = 0.018) and the oval subtype (adjusted *P* = 0.008). However, because the sample sizes of W-shaped and V-shaped groups were small, these results may require a larger sample size to support them.

The present study has several limitations. Firstly, this study was a retrospective, cross-sectional and single center design, which may lead to selection bias, residual confounding and cannot determine temporal or causal relationships. Secondly, the sample of eyes without PS and the rarity of the W-shaped and V-shaped subtypes limited the precision of analyzing the axial length and PS morphology. Thirdly, isolated inner or outer retinoschisis was relatively rare in this cohort, making it difficult to evaluate the internal tractional and external factors. BCVA may also have been influenced by coexisting MH or CNV. Finally, B-scan ultrasonography provides only sectional information and has a lower resolution than ultra-widefield OCT. Therefore, it may not represent the three-dimensional configuration of PS, and shallow or subtle PS may have been underdiagnosed [36]. In addition, the spatial relationship between PS and the macula was not systematically evaluated in this retrospective study. Further research is warranted to elucidate the complex pathogenesis of MRS by prospective cohort investigations with large sample sizes combined with three-dimensional ocular reconstruction.

## Conclusions

In conclusion, ERM was commonly observed in eyes with MRS and seemed to be independently associated with MRS development. Posterior staphyloma morphology in MRS patients has diverse characteristics.

## Data Availability

All data generated or analyzed during this study are included in this published article.

## Abbreviations

MRS: Myopic macular retinoschisis
D: Diopters
MH: Macular hole
OCT: Optical coherence tomography
PS: Posterior staphyloma
ERM: Epiretinal membrane
RPE: Retinal pigment epithelial
BCVA: Best corrected visual acuity
CRT: Central retinal thickness
SD: Standard deviation
IQRs: Interquartile ranges
GEE: Generalized estimating equation
OR: Odds ratio
CI: Confidence intervals
FD: Foveal detachment
CNV: Choroidal neovascularization
3D MRI: Three-dimensional magnetic resonance imaging
